# Shared cerebral metabolic pathology in non-transgenic animal models of Alzheimer's and Parkinson's disease

**DOI:** 10.1007/s00702-020-02152-8

**Published:** 2020-02-06

**Authors:** Jelena Osmanovic Barilar, Ana Knezovic, Ana Babic Perhoc, Jan Homolak, Peter Riederer, Melita Salkovic-Petrisic

**Affiliations:** 1 Department of Pharmacology, University of Zagreb School of Medicine, Salata 11, 10 000 Zagreb, Croatia; 2 Center of Mental Health, Department of Psychiatry, Psychosomatics and Psychotherapy, University Hospital, Würzburg, Füchsleinstrasse 15, 97080 Würzburg, Germany; 3 Department and Research Unit of Psychiatry, Institute of Clinical Research, University of Southern Denmark, Odense, Denmark; 4 Institute of Fundamental Clinical and Translational Neuroscience, Research Centre of Excellence, Croatian Institute for Brain Research, University of Zagreb School of Medicine, Salata 12, 10 000 Zagreb, Croatia

**Keywords:** Parkinson’s disease, Alzheimer’s disease, Non-transgenic animal models, Insulin resistant brain state, Cerebral glucose metabolism

## Abstract

Parkinson’s disease (PD) and Alzheimer’s disease (AD) are the most common chronic neurodegenerative disorders, characterized by motoric dysfunction or cognitive decline in the early stage, respectively, but often by both symptoms in the advanced stage. Among underlying molecular pathologies that PD and AD patients have in common, more attention is recently paid to the central metabolic dysfunction presented as insulin resistant brain state (IRBS) and altered cerebral glucose metabolism, both also explored in animal models of these diseases. This review aims to compare IRBS and alterations in cerebral glucose metabolism in representative non-transgenic animal PD and AD models. The comparison is based on the selectivity of the neurotoxins which cause experimental PD and AD, towards the cellular membrane and intracellular molecular targets as well as towards the selective neurons/non-neuronal cells, and the particular brain regions. Mitochondrial damage and co-expression of insulin receptors, glucose transporter-2 and dopamine transporter on the membrane of particular neurons as well as astrocytes seem to be the key points which are further discussed in a context of alterations in insulin signalling in the brain and its interaction with dopaminergic transmission, particularly regarding the time frame of the experimental AD/PD pathology appearance and the correlation with cognitive and motor symptoms. Such a perspective provides evidence on IRBS being a common underlying metabolic pathology and a contributor to neurodegenerative processes in representative non-transgenic animal PD and AD models, instead of being a direct cause of a particular neurodegenerative disorder.

## Introduction

Parkinson’s disease (PD) and Alzheimer’s disease (AD) are the most common chronic neurodegenerative disorders. PD is characterized by classic motor dysfunction symptoms due largely to the loss of dopaminergic neurons in the substantia nigra pars compacta (SNpc) and intracytoplasmic aggregates of the presynaptic protein α-synuclein (α-Syn) in formations called Lewy bodies (Davie 2008). On the other hand, AD is characterized by cognitive decline due to neuronal loss in the hippocampus (HPC) and temporal cortex, associated with pathological accumulation of aggregates of amyloid beta protein (Aβ) which forms extracellular plaques, and hyperphosphorylated tau protein which forms intracellular neurofibrillary tangles (Ballard et al. 2011). However, in the late stage of PD, the majority of patients also manifest cognitive deficits and dementia (Caballol et al. 2007; Gratwicke et al. 2018), while motor symptoms which also include parkinsonism are present in the majority of patients with end stage AD (O'Keeffe et al. 1996; Ballard et al. 2011; Levin 2019). Despite different phenotypes, AD and PD share some common pathological features, e.g. oxidative stress and neuroinflammation (Han et al. 2018). Metabolic changes presented as insulin resistant brain state (IRBS) have recently gained more and more attraction as the pathophysiological core of AD (Hoyer 2004; Chen et al. 2014; Defelice and Ferreira 2002; de la Monte et al. 2014), particularly after the results of epidemiological studies demonstrating that type 2 diabetes mellitus (DM) is a risk factor for AD (Santiago and Potashkin 2013). However, some studies provided evidence that IRBS is also occurring in PD patients (Athauda and Foltynie 2016; Yang et al. 2018) and that DM is a risk factor for PD as well (Biosa et al. 2018). Consequently, clinical trials have been designed to test the therapeutic potential of antidiabetic drugs, in particular intranasal insulin (Claxton et al. 2015) and incretins (glucagon-like peptide-1 [GLP-1] analogues) in AD and PD patients (Athauda and Foltynie 2016; Femminella and Edison 2014). AD and PD seem like two streets going in parallel to the same direction called neurodegeneration with some overlap in their paths, and the nature of this overlap and the point at which it starts in the course of their pathophysiology seem to be the crucial questions.

The pathology in both AD and PD starts years before the first clinical symptoms and is difficult to trace in living humans, which is why animal PD and AD models are valuable tools. Although one should keep in mind that none of the animal models is able to fully recapitulate PD and AD pathology and symptoms, they are currently the only tool available to study the underlying pathophysiology mechanisms of these two diseases. Based on the involvement of gene manipulation in designing of the models, they can be classified in transgenic (genetic) and non-transgenic models generated by injection of different chemical, mainly neurotoxic compounds. Each group has advantages and disadvantages, but genetic models generally represent the early onset forms of familial AD and PD, while the prevailing majority of AD and PD patients suffer from the sporadic and idiopathic forms of these diseases, respectively, for which non-transgenic animal models are more appropriate. This review aims to point out the similarities and differences in IRBS and related cerebral metabolic pathology between the non-transgenic PD and AD animal models. The representative non-transgenic AD and PD models will be compared from a perspective of similarities in the intracellular mechanism and target(s) of the toxicity of the compounds used to construct the models as well as the principles of their selectivity toward targeted neurons and non-neuronal cells. Finally, the alterations in insulin brain system and related interaction between insulinergic and dopaminergic transmission, particularly regarding the time frame of AD/PD pathology appearance in correlation with cognitive and motor symptoms will be pointed out.

## Direct or indirect toxicity as a cause of the central insulin-related metabolic pathology

Chemically induced non-transgenic animal models of AD and PD play an important role in defining critical disease-related mechanisms. Peripheral or central application of different substances, sometimes even in different parts of the brain (e.g. by intracerebroventricular [icv] or intrastriatal injection), with different mechanisms of action, results in different clinical symptoms which mimic those predominant in AD or PD patients. However, despite all these differences, these models also point to the common underlying molecular mechanisms of neuronal dysfunction (e.g. neuroinflammation, mitochondrial dysfunction and oxidative stress) when neurons and surrounding microglia and astrocytes are in stress following damage induced first by injection trauma and consequently by toxin-induced chemical reactions. It is still not clear whether neuroinflammation is the driven force of neurodegenerative disorders or is it a consequence of the metabolic dysfunction occurring earlier in the progression of diseases (Yin 2016). Based on its chemical structure, streptozotocin (STZ) might play multiple roles as a nonspecific cytotoxic compound which causes mitochondrial dysfunction, oxidative stress and neuroinflammation, as well as a selective metabolic toxin which causes damage to insulin-producing/secreting and insulin receptor (IR)-expressing cells (Correia et al. 2013; Eleazu et al. 2013). On the other side, 6-hydroxydopamine (6-OHDA) also demonstrates nonspecific toxicity by causing oxidative and mitochondrial damage and neuroinflammation, as well as selective toxicity to the dopaminergic neurons (Walsh et al. 2011). Despite of that, both e.g. STZ/AD and 6-OHDA/PD models seem to share IRBS and other insulin-related pathological features to a certain extent.

### Parkinson’s disease models

Although there are several neurotoxin-generated animal models of PD, the two golden standards are rats treated with 6-OHDA, and mice (or primates) treated with 1-methyl-4-phenyl-1,2,3,6-tetrahydropyridine (MPTP) (Tieu 2011) which will be more closely elaborated in this review.

#### Molecular targets responsible for selective toxicity

MPTP and 6-OHDA share the same molecular target both at the cellular membrane (i.e. dopamine transporter [DAT], a target for selectivity, but not the direct toxicity) and intracellularly (mitochondria, the target of toxicity). The MPTP model is usually generated by a peripheral (subcutaneous, intraperitoneal) administration of MPTP (intranasal application has been recently introduced as well) which crosses the blood–brain barrier and is converted by monoamine oxidase-B to MPP+  in astrocytes, so it is MPP+  which is then a selective substrate for the high affinity DAT at the dopaminergic neuron axon terminals (Storch et al. 2004). Inside the dopaminergic neuron, MPP+  is actively transported into mitochondria where it interferes with the respiratory chain and inhibits complex I, inducing energy depletion and reactive oxygen species (ROS) production (Przedborski 2000). Since 6-OHDA does not cross the blood–brain barrier, the 6-OHDA model is generated by a central, stereotaxic administration (usually intrastriatal, but application to other regions is also possible) of 6-OHDA, which, as a hydroxylated analogue of dopamine, is also a selective substrate for DAT (Storch et al. 2004). Once inside the cell, it accumulates in the cytosol and is readily oxidized leading to ROS generation and ultimately, oxidative stress-related cytotoxicity and mitochondrial fragmentation (Walsh et al. 2011; Solesio et al. 2013).

#### Selective neuronal and regional toxicity

DAT is selectively located at the dopaminergic neuron axon terminals concentrated in several brain regions, which leads to the prevailing selective accumulation of MPP+  and 6-OHDA in the nigrostriatal DAT-expressing dopaminergic neurons, particularly in the SNpc, found heavily involved in motor function but also in learned responses to stimuli (Da Cunha 2006). Dysfunctional mitochondria-based lesions in this region lead to primarily motor deficits but could also induce learning deficits similar to those found in PD patients (Da Cunha 2003). Intrastriatal 6-OHDA administration leads to depletion of catecholamine-expressing neurons and a selective inhibitor of noradrenergic transporter is generally used for protection of noradrenergic neurons, but even then, to a lesser extent, 6-OHDA is still capable of damaging noradrenergic neurons (Fulceri et al. 2006). This could be, at least partly, responsible for the lesions seen also in the locus coeruleus (Bonito-Oliva et al. 2014) as well as within the nucleus of the solitary tract (Falquetto et al. 2017) and the distinct cortical regions (Becker et al. 2018), associated with both central (cognitive impairment, depression, anxiety, olfactory deficit) as well as peripheral (cardiovascular, gastrointestinal) non-motor symptoms (Bonito-Oliva et al. 2014; Falquetto et al. 2017; Becker et al. 2018; Feng et al. 2019). MPTP neurodegeneration is also not exclusively limited to the nigrostriatal region, since MPTP was shown to bind to other brain regions as well (Javitch et al. 1985), and peripheral MPTP treatment was found to induce lesions in the hypothalamus (Gibb et al. 1986) which were associated with anorexia manifested prior to the appearance of motor deficits (Sandyk et al. 1990).

Additionally, different routes of neurotoxin administration could also account for extrastriatal alterations. It has been hypothesized that in the 6-OHDA model generated by central toxin administration, peripheral alterations in the gastrointestinal tract are indirect, as a consequence of central dopaminergic denervation and involvement of efferent vagal nerves (Pellegrini et al. 2016). On the other side, in the MPTP model, designed by peripheral toxin administration, peripheral pathology in the gastrointestinal tract could be a direct effect of the toxin on peripheral neuronal circuits, which spreads to the brain by afferent vagal nerves, and, additionally, an indirect effect, which results from the consequent central dopaminergic neurodegeneration, as reviewed by Pellegrini et al. (2016). This diversity in non-motor pathology which accompanies the common striatal dopaminergic deficits in non-transgenic PD models seems to fit the human PD condition for which the work of Braak et al. (2003) indicates that the neurodegenerative process could start in the peripheral nervous system, and progress towards the central nervous system in a caudal-to-rostral direction, so called bottom-up concept of propagation. However, others argue that, based on cases with intact vagal motor nucleus, the Braak staging cannot apply to all PD cases suggesting that there are different PD endophenotypes—clinical correlates (Jellinger 2019). This suggests that the neocortex may not necessarily be the final stage of bottom-up propagation. In view of IRBS, one could speculate that loss of energy/IRBS might be a trigger that causes/facilitates cortico-striatal glutamatergic excitotoxicity with consequences like the degeneration of striatal dopaminergic synapses and α-Syn pathology, and a retrograde striatal-nigral dopaminergic dysfunction and degeneration. From this perspective, based on the finding of cortical IRBS induced in rats by chronic exogenous corticosterone administration (Osmanovic et al. 2010), it could be further hypothesized that chronic stress accompanied by IRBS, might be a clinical/behavioural basis for a top-down propagation of neurodegeneration. A research demonstrating that cortical dysfunction could be the initial top-down alteration to affect vulnerable dopaminergic neurons, i.e. that cortical abnormalities are prodromal to motor symptoms, has been reviewed by Foffani and Obeso (2018). However, looking from another perspective, it has been suggested that parallel cellular pathologies in PD would give the first symptoms in the system with the lowest functional threshold due to selective vulnerability of different cell types/regions (Engelender and Isacson 2017). In line with that, there is a selective vulnerability of different brain regions to accumulated α-Syn proposed to account for the pattern of PD progression, with nigral dopaminergic neurons being highly sensitive in this respect (Brooks 2010).

Beside neurons, direct toxicity of 6-OHDA towards astrocytes has not been documented, whereas it can occur following MPTP administration due to the conversion of MPTP into the toxic MPP+  exclusively in astrocytes (Storch et al. 2004). Indirect effects of these neurotoxins on astrocytes are being more and more explored, since a growing body of literature indicates that astrocytes have an extremely important role in both recovery and aggravation of neurodegenerative disorders, including AD and PD (Maragakis and Rohtstein 2006). Despite common features shared throughout the brain, astrocytes show region-dependent density and vulnerability to injuries (Xu et al. 2001) and differences in expressing membrane structures (e.g. ion channels, transporters, etc.) involved in signalling (Bordey and Sontheimer 2000; Saab et al. 2012). In line with that, a recent in vitro study of 6-OHDA toxicity demonstrated huge differences in survival of dopaminergic neuronal cells in the presence of midbrain astrocytes in comparison to forebrain and hindbrain astrocytes, which was found related to 6-OHDA-induced NO release and consequent triggering of brain derived neurotrophic factor (BDNF) release from the neighbouring astrocytes (Datta et al. 2018).

#### Direct toxicity towards the insulin receptor

It is elusive whether the insulin-related metabolic changes observed in these PD models are associated with the damage solely in the nigrostriatal or/and in other brain regions, and secondly, whether they are the consequence of direct toxic effects or are induced indirectly by a lack of dopamine signalling. Considering direct toxicity, literature provides no direct evidence on the neuronal co-expression of DAT and insulin receptor (IR). However, there are reports indicating an extensive co-expression of tyrosine hydroxylase (the rate-limiting enzyme for DA synthesis used as a marker for dopaminergic neurons) with IR in the ventral tegmentum and substantia nigra (Figlewicz et al. 2003). These data suggest the presence of DAT–IR co-expression in striatal neurons and allow speculation that direct toxicity towards IR with a consequent dysfunction in insulinergic signalling might occur along with the reduction in dopaminergic signalling. Additionally, from the above mentioned 6-OHDA/MPTP-affected extrastriatal regions, the cortical and hypothalamic ones have been in particular highly enriched in insulin and IR (Hill et al. 1986; Schulingkamp et al. 2000) and, thus, also susceptible to damage by these toxins. Furthermore, it has been reported that insulin sensing by hypothalamic astrocytes which have a high IR expression (Przedborski 2000), co-regulates brain glucose sensing and systemic glucose metabolism (Garcıa-Caceres et al. 2016). This indicates that neurotoxin-induced astrocyte damage due to MPP+  could be an alternative path of generating insulin-related metabolic dysfunction in the brain of MPTP-induced PD animal models.

#### Indirect toxicity towards insulinergic signalling

There are numerous reports demonstrating vulnerability of dopaminergic D1 and D2 receptors to MPTP and 6-OHDA toxicity (Falardeaur et al. 1988; Graham et al. 1990; Weihmuller et al. 1990; Woiciechowsky et al. 1995; Tanji et al. 1999; Vučković et al. 2010). In general, a significant decrease in D2 binding sites in the substantia nigra, seen up to 3 weeks post MPTP treatment in mice (Tanji et al. 1999), is then followed by a compensatory D2 receptor up-regulation which lasts for about 3 months (Weihmuller et al. 1990). On the other hand, 3 months are required for the up-regulation of D1 receptor binding sites which is still evident after 5 months (Weihmuller et al. 1990). Such a dysregulation in D1/D2 receptor expression in the nigrostriatal region may have a huge impact on the striatal insulin system since dopamine D2 receptors are modulators of insulin secretion and mice lacking these receptors display an impaired glucose metabolism (Garcia-Tornadu et al. 2010). Furthermore, striatal dopamine receptors modulate the expression of IR and insulin like growth factor-1 (IGF-1) receptor as well as glucose transporter-3 (GLUT-3) (Anitha et al. 2012). Small changes in dopamine levels in the early post- treatment phase could affect preferentially the activation of low (D1) versus high (D2) affinity receptors while the D2-mediated effects become more prominent in the advanced phase (Anitha et al. 2012). Evidence on the interplay between dopamine and insulin signalling (Kleinridders et al. 2015; Stouffer et al. 2015) suggests that neurotoxin-induced lack of dopamine is likely to result in indirect detrimental effects due to insulin dysfunction.

#### Time course of the appearance of insulin signalling dysfunction and alterations in glucose metabolism in the brain

The expression of IR and elements downstream its signalling pathway have been mostly explored in the 6-OHDA model and to a lesser extent in the MPTP model (Table 1). Both were found altered in the first week following the neurotoxin’s application, although the regions explored were different; insulin binding to IR was decreased by 25% in the arcuate nucleus 7 days after 6-OHDA treatment (Wilcox et al. 1989), while decreased phosphorylation of enzymes downstream the IR signalling (phosphatidylinositol-3 kinase [PI3K], and protein kinase B [AKT]) was found 5 days following MPTP administration in the whole mouse brain, persisting up to 2 weeks (Hu et al. 2018; Liu et al. 2018). Dysfunctional IR signalling cascade in the 6-OHDA model was reported in the striatum; reduction of PI3K and AKT phosphorylation was found after 2 (Rabie et al. 2018) and 6 weeks (Morris et al, 2008), while increased striatal IRS2 serine phosphorylation, a marker of insulin resistance, was observed after 6 weeks (Morris et al. 2008). There is no data on IR signalling in the brain in periods > 1 month following MPTP treatment (Table 1).

**Table 1:** Comparison of insulin-related pathology, mitochondrial dysfunction and oxidative stress, neuroinflammation, cognitive and motoric symptoms between the representative non-transgenic animal models of Parkinson’s and Alzheimer’s diseases

Disease and post-treatment time	Insulin signalling (insulin receptor and/or its signalling pathway)		Cerebral glucose metabolism/glucose transporters		Mitochondrial dysfunction/oxidative stress		Neuroinflammation		Cognitive impairment		Motoric dysfunction	
Models	6-OHDA	MPTP	6-OHDA	MPTP	6-OHDA	MPTP	6-OHDA	MPTP	6-OHDA	MPTP	6-OHDA	MPTP
≤ 24 h	ND	ND	ND	ND	ND	+^36^	ND	ND	ND	ND	ND	ND
> 1 day– 1 w	↓IR^1^	↓pPI3K ↓pAKT^2^	ND	≈ GLUT1^29,30^	ND	+^38^	+^9,11^	+^40,41,42^	ND	↓MWM^46,47^	↓Rotarod^15^	↓Rotarod^,35,42,44,51^ BW^38^ ≈↓OF^44,49^
> 1 w–≤ 4 w	↓pPI3K ↓pAKT^2,3^	↓pPI3K ↓pAKT^28^ ↓pGSK3^28^	↓FDG-PET ^5,6^	ND	+^16,17,20^	+^35,37,39^	+^3,4,8,10,11,12,15^	+^43,44,45^	↓MWM^21,22,23^ PA^27^	↓MWM ≈ OF^48,49,50,51^	↓Rotarod^3,4,6,11,15,16^	↓Rotarod^39,43,50^ BW^50,51^ ≈↓OF^45,49^
> 4 w–≤ 12 w	↓pPI3K/pAKT^4^ ↑pIRS2^4^ ↓pGSK3^4^	ND	↓↑FDG-PET^7,8^	↑↓FDG-PET^32^ ≈↓GLUT1^31^	+^18,19^	+^34^	+^8,13,14^	+^34,45^	↓MWM^24,25,26^ PA	↓MWM^52^	↓Rotarod^13,14,19^	↓PMRS^47^ ↓OF^34,45^
> 12 w	ND	ND	ND	↓↑FDG-PET^33^	ND	ND	ND	ND	ND	≈ MWM^53,54^	ND	↓AC, PMRS^55,47^

Disease and post-treatment time	Insulin signalling (insulin receptor and/or its signalling pathway)		Cerebral glucose metabolism/glucose transporters		Mitochondrial dysfunction/oxidative stress		Neuroinflammation		Cognitive impairment		Motoric dysfunction	
Models	STZ	Aβ	STZ	Aβ	STZ	Aβ	STZ	Aβ	STZ	Aβ	STZ	**Aβ**
≤ 24 h	↓IR^1^	ND	↑GLUT2^1^	ND	ND	ND	**+**^1^	ND	ND	ND	ND	ND
> 1 day–≤ 1 w	↓IR, ↓pIR ≈ pIRS, ↓pPI3K, ↓pGSK3^2,3,4^	ND	↓glucose neuron. uptake, ↓GLUT3, ↓GLUT1^16,17,18,19,20^	ND	ND	ND	**+**^39^	ND	ND	↓PA ↓MWM^38,40^	ND	≈ OF^42^ ≈ AC^35^
> 1 w–≤ 4 w	↓Ins mRNA, ↓↑IR mRNA, ≈ ↓IR ↑p/serIRS, ↓IDE, ↓pGSK-3^2,3,4,5,6,7,9,10,11,12^	↓pAKT^14,15^ ↓pGSK3/ ↑GSK3β^42^	↓glucose neuron. uptake, ↓FDG-PET ↓GLUT3^17,18,21,22,23,24^	↓membr/total GLUT4 ≈ GLUT1 ≈ GLUT3^14,15^	+^19,26,28,29,30,32^	+^33,34,35^	+^19,29,32^	+^36,37,38^	↓MWM PA^2,26,28,30^	↓MWM ↓PA^33,34,41,42^	↓≈ OF ≈ Rotarod^26,29,30,32^	≈ AC^34^ ↓OF^41^
> 4 w–≤ 12 w	↓Ins mRNA, ↓↑IR mRNA, ↓IDE, ≈ plasma Ins ^2,8,13,24^	ND	↓FDG-PET ↑CSFglucose ^25^	ND	+^27,31,^	ND	+^31,29^	ND	↓MWM PA^2,31^	ND	≈ OF ≈ AC^44^	ND
> 12 w	↓Ins mRNA, **↓**IR, **↓**IDE, ↑pGSK-3^2^	ND	↓FDG-PET ≈ CSFglucose ^25^	ND	ND	ND	ND	ND	↓MWM PA^2,24^	ND	≈ OF^43^	ND

References for Parkinson’s disease models: 1. Wilcox et al. (1989); 2. Hu et al. (2018); 3. Rabie et al. (2018); 4. Morris et al. (2008); 5. Silva et al. (2013); 6. Jang et al. (2012); 7. Casteels et al. (2008); 8. Shyu et al. (2009); 9. Walsh et al. (2011); 10. Mori et al. (2018); 11. Crabbé et al. (2019); 12. Cicchetti et al. (2001); 13. Goes et al. (2018); 14. Goes et al. (2014); 15. Thornton and Vink (2012); 16. Chen et al. (2018a, b); 17. Afshin-Majd et al. (2017); 18. Haddadi et al. (2018); 19. Singh et al. (2017); 20. Ma et al. (2015); 21. Grospe et al. (2018); 22. Ma et al. (2014); 23. Horita et al. (2015); 24. Nezhadi et al. (2016); 25. Ramirez-Garrcia et al. (2015); 26. Perez et al. (2009); 27. Razavinasab et al. (2013); 28. Liu et al. (2018); 29. Lagrue et al. (2010); 30. Puchades et al. (2013); 31. Sarkar et al. (2014); 32. Brownell et al. (2003); 33. Peng et al. 2016; 34. Zhang et al. (2019a, b); 35. Lim et al. (2019); 36. Sriram et al. (1997); 37. Zhu et al. (2019); 38. Krishnamoorthy et al. (2019); 39. Wang et al. (2018a, b); 40. Zhao et al. (2007); 41. Han et al. (2018); 42. Yang et al. (2018); 43. Jing et al. (2017); 44. Yang et al. (2017); 45. Churchill et al. (2017); 46. Prediger et al. (2006); 47. Vezoli et al. (2011); 48. Haga et al. (2019); 49. Castro et al. (2013); 50. Yabuki et al. (2014); 51. Moriaguchi et al. (2012); 52. Costa et al. (2014); 53. Fernandez-Ruiz et al. (1995); 54. Fifel et al. (2013); 55. Ko et al. (2016)

References for Alzheimer’s disease models: 1. Knezovic et al. (2017); 2. Barilar et al. (2015); 3. Agrawal et al. (2010); 4. Gupta et al. (2018); 5 Deng et al. (2009); 6. Grünblatt et al. (2007); 7. Lester-Coll et al. (2006); 8. Lee et al. (2014); 9. Wang et al. (2018a, b); 10. Nassar et al. (2018); 11. Salkovic-Petrisic et al. (2006); 12. Shonesy et al. (2012); 13. Knezovic et al. (2015); 14. Pearson-Leary et al. (2012); 15. Garabadu and Verma (2019); 16. Rodrigues et al. (2019); 17. Costa et al. (2016); 18. Dos Santos et al. (2018); 19. Biswas et al. (2018); 20. Deng et al. (2009); 21. Salkovic-Petrisic et al. (2014); 22. Chen et al. 2017); 23. Knezovic et al. (2018); 24. Babic-Perhoc et al. (2019); 25. Heo et al. (2011); 26. Kumar and Bansal (2018); 27. Correia et al. (2013); 28. Kumar and Singh (2017); 29. Wang et al. (2019); 30. Wei et al. (2019); 31. Jayant et al. (2016); 32. Javed et al. (2015); 33. Jafari et al. (2015); 34. Liu et al. (2014); 35. Hu et al. (2012); 36. Maione et al. (2017); 37. Budni et al. (2017); 38. Russo et al. (2012); 39. Kraska et al. (2012); 40. Yan et al. (2004); 41. Wu et al. (2007); 42. Xu et al. (2016); 43. Motzko-Soares et al. (2018); 44 Ozkay et al. (2012)

6-OHDA, 6-hydroxydopamine; MPTP, 1-methyl-4-phenyl-1,2,3,6-tetrahydropyridine; STZ, streptozotocin; Aβ, amyloid beta; h, hour; w, week; ND, no data; IR, insulin receptor; pIR, tyrosine phosphorylated IR; pPI3K-phosphorylated phosphatidylinositol-3 kinase; p/serIRS, serin-phosphorylated insulin receptor substrate; pGSK-3, phosphorylated glycogen synthase kinase; IDE, insulin degrading enzyme; GLUT, glucose transporter; FDG-PET, 2-deoxy-2-[fluorine-18]fluoro-d-glucose-Positron emission tomography; CSF, cerebrospinal fluid; neuron, neuronal; membr, membrane; PMRS, Parkinsonian Monkey Rating Scale; MWM, Morris Wather Maze test; PA, Passive Avoidance test; OF, Open field; Ins, insulin; AC, active cages; ↓, significantly decreased; ↑, significantly increased; ≈, no significant changes; +, reports on significant changes of mitochondrial dysfunction and oxidative stress, and neuroinflammation

Changes in cerebral glucose metabolism, measured by ^18^F-fluorodeoxyglucose positron emission tomography (FDG-PET) were detected in 6-OHDA and MPTP models. The earliest time-point explored following neurotoxin treatment was 4 weeks after 6-OHDA application in rats, demonstrating decreased glucose uptake in the basal ganglia, olfactory bulb, primary motor cortex, substantia nigra, and tegmental nucleus (Jang et al. 2012; Silva et al. 2013). The finding of increased glucose uptake in the somatosensory cortex and ventral caudate-putamen in the same experiment indicates that alterations in cerebral glucose metabolism following 6-OHDA treatment are region-specific (Jang et al. 2012), as supported also by a research of Shyu et al. (2009). Another study in rats performed FDG-PET analysis 6–11 weeks following 6-OHDA treatment and demonstrated decreased glucose metabolism in the sensory-motor cortex and hippocampus, revealing also a positive correlation between the degree of DAT impairment and the change in hippocampal glucose metabolism (Casteels et al. 2008). FDG-PET studies performed in non-human primates (macaques) several months (exact time not specified) after MPTP administration, reported increased glucose uptake in the putamen/pallidum, thalamus, pons, medial frontal gyrus/cingulate and sensorimotor cortex, and decreased glucose uptake in the posterior parieto-occipital cortex (Peng et al. 2016). Another study in macaques reported glucose metabolism to be decreased in the caudate, putamen, thalamus and primary motor cortex (range 35–50%) and enhanced in the globus pallidus by 15%, as measured 2–3 months post MPTP treatment (Brownell et al. 2003). These changes were accompanied by a decreased DAT content in the putamen and caudate (60–65%) and no changes in D2 receptor level (Brownell et al. 2003). Glucose transporters play a key role in the regulation of cellular glucose uptake, but their research in these two PD models has been very modest with only few studies available on this topic in mice MPTP models (Table 1); two studies reported no changes in cerebral GLUT1 level in the acute phase (Lagrue et al. 2010; Puchades et al. 2013), while a study of Sarkar et al. (2014) demonstrated decreased GLUT1 expression in the striatum accompanied by decreased expression of DAT in the caudatus/putamen and the substantia nigra regions 5 weeks following MPTP treatment.

### Alzheimer’s disease models

Unlike PD modelling, there is no golden standard in non-transgenic AD models due to the predominant use of transgenic mice models. However, among the chemically induced models, the two most frequently used are those with central administration of streptozotocin (STZ-icv) or Aβ_1-42_, but the former has been far more extensively characterised (Table 1) (Kamat 2015; Facchinetti et al. 2018).

#### Molecular targets of selective toxicity

STZ is a selective substrate for the glucose transporter-2 (GLUT2) (Schnedl et al. 1994) which itself is also a target molecule for STZ toxicity (Gai et al. 2004). Exclusive selectivity of STZ for the GLUT2 isoform was explored generally in comparison to the GLUT1 isoform and not the other GLUTs, so one should not exclude STZ-icv toxicity towards GLUT3 whose expression was found decreased in the STZ-icv rat model (Salkovic-Petrisic et al. 2014) as well as the hippocampal neuronal stem cells in vitro (Sun et al. 2018).

Unlike STZ, exogenous (similar to endogenous) Aβ peptide fragments seem to lack selectivity towards membrane molecular targets since the intracellular intake of Aβ peptides is not operated via a selective transporter. Instead, three different pathways of its internalization have been proposed; for both Aβ_1–40_ and Aβ_1–42_ endocytic mechanisms seem to be the major ones although both could enter a neuron via the pore-forming protein perforin, while only Aβ_1–42_ was shown to enter via the receptor for advanced glycation end products (RAGE) (Lana et al. 2017). Endocytosis is two times more efficient for soluble Aβ_1–42_ than for soluble Aβ_1–40_ but both are predominantly taken up via clathrin- and dynamin-independent mechanisms (Wesen et al. 2017).

Regarding intracellular toxicity, both STZ and Aβ fragments target the mitochondria (Correia et al, 2013; Narayan et al. 2014) but as an N-nitroso compound, STZ acts like an alkylating agent which damages DNA leading to mitochondrial dysfunction (Bolzán and Bianchi 2002). A massive production of ROS and nitric oxide, as well as decreased levels of ATP and glutathione (GSH) in the mice hippocampus were found already 24 h following STZ-icv administration of a 2 mg/kg dose (Amiri et al. 2017). Five weeks following a single bilateral STZ-icv administration of 3 mg/kg dose to rats, a decrease in the mitochondrial transmembrane potential, repolarization level, ATP content, respiratory state 3, respiratory control ratio and ADP/O index, and an increase in the lag phase of repolarization were observed (Correia et al. 2013). These changes were accompanied by a decrease in pyruvate and α-ketoglutarate dehydrogenases and cytochrome c oxidase activities, and an increase in the susceptibility to calcium-induced mitochondrial permeability transition was found as well (Correia et al. 2013). Mitochondria are also intracellular targets of Aβ as a consequence of their ability to form pores on the plasma membrane which then stimulate Ca^2+^ influx (Narayan et al. 2014). This leads to calcium-dependent ROS production, decrease in antioxidant GSH and also a profound increase in astrocytes’ NADPH oxidase, resulting in induction of mitochondrial depolarization/deregulation (Angelova and Abramov 2016). Aβ acts preferentially on astrocytes but causes neuronal death within 24 h. (Abramov and Duchen 2005).

#### Selective neuronal and regional toxicity

It is common knowledge that STZ is toxic to insulin-producing cells and damages the function of IR-expressing cells when given peripherally (Szkudelski 2001; Eleazu et al. 2013). Both types of cells are expressed in the brain (Plum et al. 2005) and are, therefore, assumed to be the targets of STZ administered icv (Salkovic-Petrisic et al. 2009; Kamat 2015). However, direct evidence of STZ entering into such cells is still lacking. The selectivity towards insulin-producing cells is based on the presence of membrane GLUT2 for which STZ is a selective substrate as mentioned above (Plum et al. 2005). GLUT2 is moderately expressed in the brain in a region-dependent manner, both in neurons and astrocytes (Arluison et al. 2004). Neuronal GLUT2 expression is possibly involved in glucose sensing, particularly in the hypothalamus, while in the hippocampus GLUT2 is supposed to participates in the regulation of neurotransmitter release and synaptic activity (Arluison et al. 2004; Jurcovicova 2014). STZ toxicity towards IR-expressing cells is not so clear at the periphery nor in the brain. One possibility might be the co-expression of GLUT2 and IR which has been demonstrated in the rat parieto-temporal cortex and hippocampus where most of the positive IR immunoreactivity was observed on the neuronal membranes, as shown by IR-NeuN co-localisation (Knezovic et al. 2017). Additionally, GLUT2–IR co-expression was demonstrated in the rat ependymal lining cells of the third ventricle in the hypothalamic region (Knezovic et al. 2017). Both GLUT2 and IR expression in these cells was found affected (increased and decreased, respectively) 1 h after STZ-icv application, providing thus an indirect evidence that GLUT2- and/or IR-expressing cells are the targets of STZ-icv toxic activity (Knezovic et al. 2017). Direct evidence of such a co-expression in other cells/regions is lacking so far but it seems very likely to occur in astrocytes (Dwyer et al. 2002; Plum et al. 2005). Similar to MPTP-induced effects, an in vitro study showed that STZ exerts a decrease in ATP level and decreased mitochondrial membrane potential not only in neurons but to a similar extent in astrocytes as well (Biswas et al. 2017). Furthermore, STZ in vitro treatment in rat astrocytes cell lines induced a significant decrease in IR mRNA and protein expression as well as a decrease in consequent phosphorylation of IRS-1, AKT, GSK-3α and GSK-3β (Table 1), further followed by increased protein expression in amyloid precursor protein (APP), beta secretase-1 (BACE1), and Aβ1–42 in astrocytes (Rajasekar et al. 2014).

Additionally, GLUT3-expressing neurons can be affected by STZ as mentioned above. Neuronal GLUT3, which is responsible for glucose transport into neurons (Simpson et al. 2008), and seems to also be a target of STZ toxicity (Salkovic-Petrisic et al. 2013), is found in the brain regions with dense neuronal synapses; the highest abundance in the rat brain is observed in the hippocampal and hypothalamic regions as well as in some layers of the cerebellum followed by high abundance in neuropil of some cerebral cortex layers, and substantia nigra (Gerhart et al. 1995). Glia cells do not express GLUT3 (Mantych et al. 1992). GLUT3 and IR co-expression has been confirmed in rat hypothalamic neurons (Kang et al. 2004), while there are no data on GLUT3 and GLUT2 co-expression in the brain.

Insulin signalling-related proteins, including IR, IRS-1, AKT, glycogen synthase kinase-3β (GSK3β) and insulin degrading enzyme (IDE), were found co-expressed with choline acetyltransferase (ChAT) in the rodent brain (i.e. in hippocampal neurons), clearly indicating insulin signalling in cholinergic neurons (Wang et al. 2009). It is, therefore, important to point out that the density of ChAT-positive cells is significantly decreased in the rat hippocampus as well as in the basal forebrain, 3 weeks following STZ-icv treatment (Majkutewicz et al. 2016). Further research is needed to elucidate whether this is indeed a direct toxic effect of STZ on the cholinergic neurons in the region crucial for cognition. Furthermore, based on the hypothesis of a different pattern of neuronal loss in the subregions of the nucleus basalis of Meynert in the AD and PD condition (Liu et al. 2015), it would be worthy to compare the non-transgenic AD and PD models in this respect.

The problem with the construction of an Aβ model lays in the fact that different Aβ sequences (mostly Aβ_1–42_ but other, shorter ones like Aβ_25–35_ can be used as well) can be administered in different forms (soluble, fibrillary) either as a single injection into target areas of the hippocampus, cerebral cortex or basal forebrain nuclei, or as prolonged intracerebroventricular perfusion by osmotic minipumps, which can then lead to different effects (Harkany et al. 1999). At the current level of knowledge, it is not possible to determine which particular neurons are selectively targeted by exogenously administered Aβ fragments. Furthermore, soluble Aβ was shown to compete with apoE for binding to the low-density lipoprotein receptor-related protein 1 (LRP1) on astrocytes (Verghese et al. 2013), indicating the possible mechanism of Aβ intake into astrocytes. Aβ-induced formation of pores for stimulation of Ca^2+^ influx with consequent ROS production and mitochondrial damage happens only on the plasma membrane of astrocytes but not in neurons (Narayan et al. 2014).

Another approach to elucidate regional Aβ targeted toxicity comes from preliminary experiments with peripheral Aβ administration in transgenic AD mice, aimed to assess its influence on the inherited Aβ pathology and also to study Aβ seeding activity (Eisele et al. 2009, 2010, 2014). In lack of literature data on non-transgenic AD models, following Aβ spreading in transgenic mice models (which are not a focus of this review) might provide valuable information. The first experiments of Eisele et al. (2009) revealed that oral, intravenous, intraocular and intranasal administration of Aβ-containing extracts from aged APP23 transgenic mice (as well as from human AD brain in case of oral administration) resulted in no detectable induction of cerebral β-amyloidosis in APP23 transgenic mice as checked 3 or 6 months later. In parallel, central administration of these Aβ extracts into different brain regions induced Aβ deposition starting in the proximity of the injection site (3 months) and further spread into adjacent brain areas (6 months), suggesting that spreading can occur along fibre tracts (Eisele et al. 2009). Unlike the injection site-dependent parenchymal deposits, induction of Aβ deposition in the vasculature was independent of the injection site (Eisele et al. 2009). Further research of the same group indicated that intraperitoneal application of transgenic mice Aβ extracts to APP23 transgenic mice requires a higher concentration and a longer incubation interval to affect Aβ deposition in the brain, compared to intracerebral administration (Eisele et al. 2010, 2014). This was manifested by formation of larger plaques and a substantially higher number of smaller plaques in the brain with no evidence for peripheral amyloid formation (Eisele et al. 2014). Interestingly, in the same set of experiments, peritoneal inoculation induced plaque seeding in all cortical regions without preferential sites, while intracerebral Aβ extract administration induced it locally at the site of injection (Eisele et al. 2014). Following a single intravenous injection of Aβ (isolated from AD patient’s brain) to APP/PS1 transgenic mice, the observed targets were only blood vessels as only cerebral amyloid angiopathy could have been observed within 180 days post injection, while amyloid plaque loads seemed unaffected (Burwinkel et al. 2018). Interestingly, in Aβ-treated transgenic mice pronounced vascular amyloid deposition was found in the thalamus region, which was not seen either after the treatment with negative control extracts, nor in intact transgenic mice (Burwinkel et al. 2018). These findings provide further support to the hypothesis that Aβ most probably lacks a selective molecular target at the cellular/neuronal membrane and that further Aβ pathology dissemination is very much dependent on the Aβ entrance site.

#### Direct toxicity towards dopaminergic neurons

Considering the AD-PD pathology interplay, and assuming that GLUT2 cell-expression is a toxicity target, it could be expected that there is a co-expression of GLUT2 and DAT, but there are no direct evidence for this in ex vivo histology analysis. Some rather indirect and elusive indication that such a co-existence should not be completely excluded comes from the in vitro study on the catecholaminergic-containing N2a cells which express GLUT2, and whose upregulation leads to increased release of dopamine (Wu et al. 2014). However, an extensive co-expression of tyrosine hydroxylase with IR in the ventral tegmentum and substantia nigra (Figlewicz et al. 2003) might suggest a direct toxicity of STZ towards the dopaminergic neurons as a base for PD-like pathology in the STZ-icv sAD model (Table 1).

No clear conclusion can be drawn for a direct toxicity of Aβ towards dopaminergic neurons in the Aβ model but such a possibility cannot be excluded merely due to a lack of evidence for Aβ selective toxicity towards particular cellular membrane structures.

#### Indirect toxicity towards dopaminergic signalling

Neurochemical analyses point to the changes in the dopaminergic neurotransmission in the brain of STZ-icv rat model observed 1 week after treatment with a low STZ-icv dose (0.5 mg/kg) as a decrease in the striatal dopaminergic D1 receptor density with no effect on D2 receptors (Šalković et al. 1995). This might be in line with timely different sensitivity of striatal D1 and D2 receptors on toxin treatment (Anitha et al. 2012). Changes in DA transmission in the STZ-icv rat model seem to be region-specific and STZ-dose- and post-treatment-dependent; an increase in DA content was observed after 1 week in the whole brain only with doses > 5 mg/kg (Lacković and Šalković 1990) while after 3 weeks, a huge decrease in DA content was reported in the hippocampus with a dose of 3 mg/kg (Arora and Deshmukh 2017; Kaundal et al. 2018) whereas at the same time point no change in DA content was seen in the striatum with a dose of 0.5 mg/kg (Ding et al. 1992). Furthermore, the expression of DAT mRNA gradually decreases in the course of post STZ-icv treatment time (0.5 mg/kg) in the ventral medial bundle reaching the level of statistical significance 4 weeks after the treatment, while, during that time it remained unchanged in the arcuate nucleus (Salković-Petrisić and Lacković 2003).

Research on the dopaminergic transmission in Aβ models indicates changes in the early post-treatment phase as shown by a study of Mukhin et al. (2019) in rats treated icv with Aβ_25–35_ fragment in which the ability of neurons in the substantia nigra to secrete dopamine in the anterior dorsomedial striatum was reduced already during the hour following the moment of administration. Similar reduction in dopamine levels was observed in the rat prefrontal cortex 2 and 48 h following the icv injection of Aβ_1–42_ (Trabace et al. 2007), and in the mice striatum 2 weeks after the Aβ_1–42_ treatment (Pandey et al. 2016).

#### Time course of the appearance of insulin signalling dysfunction and alterations in glucose metabolism in the brain

Considering the selective toxicity of STZ towards insulin-producing/secreting cells, it is to be expected that insulin signalling dysfunction has been more extensively explored in the STZ-icv model compared to the Aβ model (Table 1). The earliest reported time-point of determination of IR expression was 1 h following STZ-icv administration of a single 1.5 mg/kg dose; mild decrease was found in IR immunoreactivity (mostly on neuronal membranes) in the parietal cortex while no changes were found in the temporal cortex and hippocampus (Knezovic et al. 2017). Additionally, decreased IR expression was found after 1 h also in the ependymal lining cells of the 3rd ventricle in the hypothalamic region (Knezovic et al. 2017). Decreased IR expression in the rodents’ hippocampus was more or less a consistent finding in the time-course of 6 months following STZ-icv treatment (Agrawal et al. 2010; Barilar et al. 2015; Gupta et al. 2018). However, no changes in IR expression was found 3 weeks after STZ-icv administration when analysis was done in homogenates of the whole cerebrum or cerebellum (Deng et al. 2009), indicating that changes are region-specific and, depending on the degree of change, might be lost when detecting in the brain as a whole. It is important to mention that changes were also observed at the gene level, seen as acute increase in IR mRNA after 2 weeks followed by a decrease later on at 9-month follow up (Barilar et al. 2015). In other studies, decreased IR mRNA expression was seen after 4 weeks not only in the hippocampus but also in the cortex, striatum and cerebellum (Gupta et al. 2018), and after 3 months in the frontoparietal cortex (Grünblatt et al. 2007). Additionally, in vitro studies showed that STZ decreases expression of both IR protein and mRNA in astrocytes (Rajasekar et al. 2014).

Looking upward of the IR signalling to the level and synthesis of insulin in the rat brain, which was found to occur in neurons and not in glia (Schechter et al. 1988), the earliest time-point of insulin mRNA assessment was 2 weeks following intracerebral STZ administration to young rat pups, in which decreased insulin gene expression was observed in the temporal lobe (Lester-Coll et al. 2006) (Table 1). In adult rats, insulin mRNA expression in the hippocampus was found unchanged after 2 and 4 weeks, but was significantly decreased in periods > 1 month following STZ-icv treatment (Barilar et al. 2015), which could be related to a lesser sensitivity of the mature neurons to STZ toxicity (Isaev et al. 2018). Decreased insulin mRNA expression was also observed in different brain regions of monkeys, including the frontal cortex and hippocampus, 5 months following STZ-icv administration (Lee et al. 2014). Considering the elements downstream the IR signalling cascade in the STZ-icv model, dysfunctionality was observed rather consistently across the studies of different research groups and at different time-points from 1 week up to 9 months post-STZ-icv treatment (Table 1). Different parameters ranging from decreased tyrosine phosphorylation of IR, through serine phosphorylation of IRS1, decreased PI3K and AKT phosphorylation, to consequently increased GSK3 activity, were explored and demonstrated starting from 1 week onward following STZ-icv treatment (Table 1) (Salkovic-Petrisic et al. 2006; Deng et al. 2009; Shonesy et al. 2012; Barilar et al. 2015; Gupta et al. 2018; Nassar et al. 2018; Wang et al. 2018a, b). A 9-month follow up study indicated that neurochemical changes related to insulinergic signalling in the hippocampus, including the alterations in IDE and gene expression, seem to follow a biphasic pattern (Knezovic et al. 2015; Barilar et al. 2015); an acute response emphasized up to 1 month post-treatment, followed by a slow progressive chronic phase seen at > 3 month time-points, which positively correlated with cognitive impairment (Knezovic et al. 2015). Insulin levels measured in cerebrospinal fluid (CSF) and plasma showed some mild variations seen only in the acute phase (Babic Perhoc et al. 2019).

Research on insulin signalling dysfunction in the brain of the Aβ_1–42_ model is modest, revealing decreased phosphorylation of AKT in the rat hippocampus 2 weeks following Aβ_1–42_ treatment (Table 1) (Pearson-Leary et al. 2012; Garabadu and Verma 2019).

STZ and Aβ_1–42_ models also display changes in cerebral glucose metabolism detected by FDG-PET and glucose uptake studies as well as by analysis of GLUTs, which were all more extensively explored in the STZ-icv model (Table 1). The earliest time-point measured was 1 h after a single dose of STZ-icv when an increase of GLUT2 expression was found in the hippocampus and hypothalamus with no changes in cortical regions (Knezovic et al. 2017). The next time-point was 48 h after the treatment when decreased glucose uptake by neurons was recorded (Rodrigues et al. 2019). In the course of time, changes in neuronal glucose uptake showed persistent decrease after 1 and 4 weeks (Costa et al. 2016; Dos Santos et al. 2018). FDG-PET studies in rats detected glucose hypometabolism starting from 3 weeks following STZ-icv treatment onward (Chen et al. 2018a, b; Knezovic et al. 2018; Babic Perhoc et al. 2019), which was observed also in STZ-icv treated monkeys after 6 and 12 weeks (Heo et al. 2011). Regarding the alterations of GLUTs, consistent findings on decreased levels of GLUT1 and GLUT3 were detected in the brain of the STZ-icv rat model starting from 1 week following treatment (Table 1) (Deng et al. 2009; Biswas et al. 2018). GLUT3 was found additionally decreased 1 month after STZ treatment (Table 1) (Salkovic-Petrisic et al. 2014).

First alterations of cerebral glucose metabolism in the Aβ1–42 model were recorded 2 weeks following the treatment when decreased levels of glucose were detected in hippocampal neurons and increased concentration was observed in the cerebrospinal fluid (Table 1) (Pearson-Leary et al. 2012). The same study found no changes in GLUT1 and GLUT3 expression in the hippocampus but translocation of the insulin-sensitive GLUT4 transporter from the intracellular compartment to the membrane was decreased (Pearson-Leary et al. 2012), as reported also by Garabadu and Verma (2019).

## Insulin and dopamine signalling interplay in non-transgenic ad and PD models

**Fig. 1:**
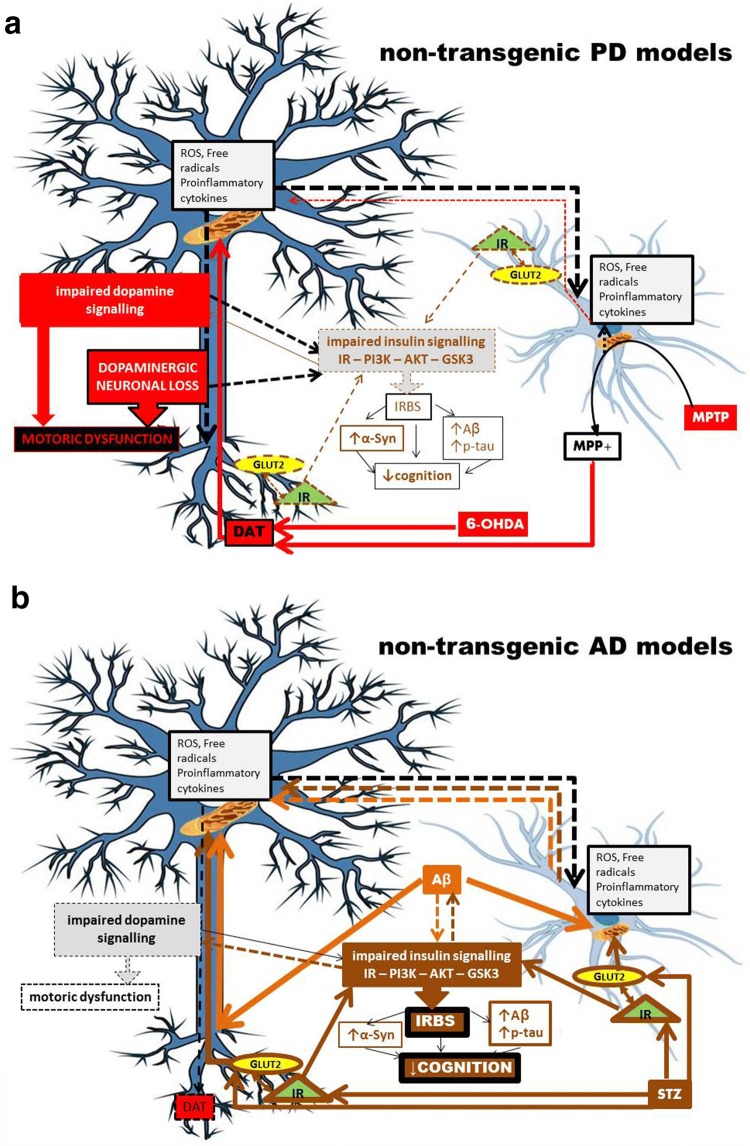
Proposed mechanism of central insulin resistance as a common pathological feature in non-transgenic models of Parkinson's and Alzheimer's disease. In 6-OHDA- and MPTP-induced PD models (**a**) impaired dopaminergic signalling is the predominating severe pathological event due to the selectivity of these compounds for entering DAT-expressing dopaminergic neurons. The extent of dopaminergic signalling impairment accompanied by dopaminergic neuronal loss (particularly in the substantia nigra pars compacta) is large enough to cause motoric dysfunction. The molecular mechanism(s) of toxicity is related to mitochondrial damage, generation of oxidative stress and proinflammatory cytokines which may further damage the respective neuron but also the neighbouring astrocytes. However, due to the dopamine—insulin interaction based on the co-expression of their major signalling parameters, disturbed dopamine signalling may be, to a lesser extent, transduced to insulin signalling downstream the insulin receptor pathway, as a secondary, collateral damage. The resulting insulin resistance may further lead to cognitive impairment, but also to the accumulation of Alzheimer’s (amyloid beta and hyperphosphorylated tau protein) and Parkinson’s (alpha-synuclein) pathological hallmarks. In the condition generated by STZ administration which induces Alzheimer’s disease (**b**), the insulin receptor and its signalling pathway are the primary, direct targets which lead to the insulin resistance state as a predominant pathological effect, both in neurons and neighbouring astrocytes expressing the targets. Therefore, it is to be expected that the extent of insulinergic signalling impairment is large (larger than induced by 6-OHDA/MPTP), because cognitive decline is pronounced, associated with pathological accumulation of the respective misfolded proteins. Damaged astrocytes can further destroy neighbouring neurons and vice versa, STZ-induced mitochondrial damage in the neuron can affect neighbouring astrocytes. In this scenario, due to the insulin–dopamine interplay, impaired insulin signalling can be transduced to dopaminergic signalling as a secondary, collateral damage (without severe dopaminergic neuronal loss), which can possibly be reflected in some motoric dysfunction expressed to a much lesser extent than in PD models. *PD *Parkinson's disease, *AD* Alzheimer's disease, *6-OHDA* 6-hydroxydopamine, *MPTP* 1-methyl-4-phenyl-1,2,3,6-tetrahydropyridine, *MPP*+, 1-methyl-4-phenylpyridinium, *STZ* streptozotocin, *Aβ* amyloid β, *α-Syn* α-synuclein, *DAT* dopamine transporter, *GLUT2* glucose transporter-2, *IR* insulin receptor, *PI3K* phosphatidylinositol-3 kinase, *AKT* protein kinase B, *GSK3* glycogen synthase kinase-3, *p-tau* phospho tau protein, *ROS* reactive oxygen species, *solid line* direct effect, *dashed line* indirect effect

At the molecular level, PD (6-OHDA/MPTP)- and AD (STZ/Aβ_1–42_)-causing neurotoxins share mitochondria as the same intracellular target of their toxicity (Walsch et al. 2011), while their targets greatly differ at the cellular membrane; PD-causing neurotoxins are both linked to DAT (Storch et al. 2004), whereas AD-causing ones are either linked to GLUT2 and/or IR (STZ) (Gai et al. 2004; Knezovic et al. 2017), or have no known preferences for membrane targets (Aβ_1–42_). These differences are expected to lead to different selectivity towards particular neurons or non-neuronal cells which express DAT, GLUT2 or IR. Although at first sight this might imply that 6-OHDA/MPTP on one side, and STZ and Aβ_1–42_ each on other sides, will target different neurons, literature does not entirely support this assumption. On the contrary, it suggests that, not only some neurons seem to co-express PD- and AD-neurotoxin targets (Fieglewicz et al. 2003), but such targets co-expression is seen additionally in non-neuronal cells like astrocytes (Przedborski 2000; Arluison et al. 2004), and astrocytes may then consequently damage neighbouring neurons (Fig. 1). In this regard, it could be speculated that a difference lays in the order in which particular cell types are being damaged with three possible scenarios; (1) neurons are attacked first which consequently causes damage to the neighbouring astrocytes, (2) astrocytes are the primary targets which then injure the neighbouring neurons, (3) neurons and astrocytes can be independently damaged in parallel. Due to selective targeting of DAT at the neuronal membrane, the first scenario most likely applies to the effects induced by 6-OHDA and MPTP. The second and the third scenarios seem to apply to STZ- and Aβ_1–42_-induced toxicity. These two compounds enter the neurons but also the astrocytes which predominately express targets (STZ/GLUT2, IR) at the membrane (Dwyer et al. 2002; Arluison et al. 2004), or the toxin (Aβ_1–42_) has preference for the astrocyte’s pore-stimulated Ca^2+^ influx with consequent astrocyte mitochondrial damage (Verghese et al. 2013). Therefore, keeping in mind the astrocytes’ region-dependent density and vulnerability to injuries (Xu and Zhang 2011), as well as differently expressing membrane signalling structures (Bordey and Sontheimer 2000), this again provides plausible evidence that, besides the dose, the site of central STZ/Aβ_1–42_ application plays a key role in determining the final toxic effect.

Since early changes have not been fully characterized in AD and PD models discussed here, and because there are other pathological processes going on in AD and PD that have not been taken into account here (e.g. apoptosis and autophagy disturbances, etc.), it is difficult to draw a conclusion on the order of dysfunction appearance between the insulin and dopamine signalling and the causal relationship between the two of them. Published data indicates that both insulin and dopamine signalling have been impaired in AD models during the first hour following neurotoxin administration; IR expression is decreased in the cortex and hypothalamus, and accompanied by increased GLUT2 expression in the hippocampus and hypothalamus of the STZ-icv model (Knezovic et al. 2017), while at the same time-point dopamine levels are found decreased in the substantia nigra in the Aβ model (Mukhin et al. 2019). Such a comparison is not possible in PD models since insulin signalling in the brain has not been explored earlier than 7 days following 6-OHDA/MPTP treatment (Wilcox et al. 1989). Additionally, from that time-point onward, impaired insulin and dopaminergic signalling in the brain (and in the striatum and hippocampus in particular) have been detected both in AD and PD models (Salkovic-Petrisic et al. 2006; Lester-Coll et al. 2006; Grünblatt et al. 2007; Morris et al. 2008; Deng et al. 2009; Agrawal et al. 2010; Shonesy et al. 2012; Lee et al. 2014; Barilar et al. 2015; Knezovic et al. 2015, 2017; Hu et al. 2018; Rabie et al. 2018; Gupta et al. 2018; Wang et al. 2018a, b; Nassar et al. 2018). On one side, there is decreased IR expression in astrocytes and neurons already 1 h after STZ application, and STZ-induced mitochondrial damage manifested during 24 h following STZ-icv treatment (Amiri et al. 2017; Knezovic et al. 2017). On the other side, insulin mRNA in adult rats cannot be detected within 2 weeks post-STZ-icv treatment (Barilar et al. 2015). Therefore, it seems likely that IR signalling is the primary pathological event following STZ-icv treatment, while a decrease in insulin synthesis comes as a secondary pathology, contributing to aggravation and progression of neurodegeneration. This hypothesis is in line with the findings of disturbed PI3K/AKT signalling pathway in neurodegeneration (as reviewed elsewhere, Rai et al. 2019). The impaired signal is further transduced to GSK3 enzyme involved in dysregulation of AD-linked Aβ homeostasis and tau hyperphosphorylation (Martinez and Perez 2013), but also in α-Syn-mediated neurodegeneration in PD (Yang et al. 2018). 6-OHDA- and MPP+ -induced neurodegeneration is associated with increased GSK3β activity also in in vitro PD models (Wu et al. 2007). All this strongly suggests that impairment in the IR signalling cascade with a consequent IRBS condition is, actually, not unique only to AD, but instead, can be considered a common underlying mechanism in neurodegenerative disorders as evidenced in non-transgenic PD and AD models (Fig. 1).

This hypothesis on the common IRBS role as a contributor to neurodegeneration in AD and PD condition strongly agrees with a recently proposed role of α-Syn in neurodegenerative disorders (Riederer et al. 2019). For a long time, α-Syn has been thought of as a specific pathological hallmark of PD. However, a growing body of evidence has emerged showing its co-localisation and synergistic effects with other proteins prone to pathological misfolding (e.g. Aβ and hyperphosphorylated tau protein) in neurodegenerative disorders other than PD, which finally led to a conclusion that α-Syn is more likely a non-specific bystander and a contributor to neurodegeneration in general, than a specific etiopathogenic factor of PD (Riederer et al. 2019).

It might be speculated that in the case of IR being the primary target of toxicity (e.g. like in the STZ-icv model) and due to the widely spread IR expression in the brain on neurons, astrocytes, endothelial and inflammatory cells, the IRBS condition and its consequences would dominate, while dopaminergic signalling dysfunction could be below the threshold for clinical manifestations, as supported by, in general, lack of significant (or only a mild) locomotor impairment in STZ-icv treated rats (Table 1, Fig. 1). Considering the important role of insulin in the brain in synaptic plasticity, learning and memory functions, such a predomination of IRBS might be reflected in cognitive impairment consistently seen in the STZ-icv model from the 2-week time point onward (Knezovic et al. 2015), and even at earlier time-points in the Aβ model (Table 1) (Schmid et al. 2017). Recent evidence suggests that the neurotoxic properties of Aβ and related cognitive impairment are mediated by oxidative stress, neuroinflammation but also by the disturbed PI3K/AKT/GSK3 signalling pathway (Morroni et al. 2016; Schmid et al. 2017; Amin et al. 2015). Additionally, decreased immunoreactivity for the vesicular acetylcholine transporter (a marker of cholinergic neurons) was observed already 1 week after Aβ fragments treatment in 6–7 weeks-old rats (Zussy et al. 2011) as well as cholinergic neuronal loss found 3 weeks following STZ-icv administration to rats (Majkutewicz et al. 2016), which could contribute to cognitive impairment in both models. Although dopamine release is acutely disturbed after Aβ fragments administration (Trabace et al. 2007), the impact of this seems not to be capable of triggering significant locomotor deficits, at least not in observational periods explored so far (Guerra de Souza et al. 2018).

Cerebral glucose metabolism, explored and found decreased already within the first post-treatment week in the STZ-icv model, and at > 1-week time-points in Aβ/AD and in both PD models (Table 1) (Chen et al. 2018a, b; Knezović et al. 2017; Babic Perhoc et al. 2019; Jang et al. 2012; Silva et al. 2013) certainly contributes to cognitive impairments seen in these models, as does the glucose hypometabolism observed in the brain of AD patients (Chen and Zong 2013). Furthermore, GLUT changes seen in these non-transgenic models are quite resembling to those found in AD/PD patients, e.g. decreased levels of GLUT3 were found in the brain of AD patients post mortem (An et al. 2018; Liu et al. 2009) accompanied by an increase in GLUT2 expression which most likely occurs in astrocytes (Liu et al. 2008).

Following administration of DAT-targeting neurotoxins, IR seems to be a collateral damage due to the evidence of IR co-expression with tyrosine hydroxylase, a marker for dopaminergic neurons (Figlewicz et al. 2003). Thus, dopaminergic loss-related signalling dysfunction would be predominant over IRBS, and evidently manifested by PD-like motoric symptoms (Table 1). However, the impact of disturbed IR signalling pathway seems to be strong enough to trigger certain cognitive impairments, as observed in 6-OHDA/MPTP models starting from 2 weeks after treatment, and onwards (Castro et al. 2013; Grospe et al. 2018).

Trans-disciplinary preclinical/clinical research indicates that insulin signalling influences dopamine availability and turnover (Kleinridders et al. 2015; Stouffer et al. 2015) and a recent post-mortem study in the brains of mentally ill patients provided evidence that the expression of dopamine signalling genes was mediated by insulin signalling genes (Mansur et al. 2019). Additionally, human data points to changes in dopaminergic system found in AD patients post-mortem; significantly lower levels of DA, its precursor and its metabolite were found in the striatum and additionally in the cingulate gyrus, amygdala and raphe nuclei (Storga et al. 1996). These reductions in DA level correlated well with the duration of AD (Pinessi et al. 1987), and decrease in DAT expression (Allard et al. 1990). Furthermore, the number of striatal dopaminergic D1/D2 receptors was found decreased in AD patients (Pizzolato et al. 1996). In line with that, it is not unexpected that AD patients develop also symptoms of motor dysfunction (O'Keeffe et al. 1996; Albers et al. 2015). It seems likely that the reasons for motor dysfunction being generally not observed in STZ/Aβ animal models of sporadic AD, could be related to a too short post-treatment observational period, and to the lack of gene-environmental interactions that have been suggested to play a role in AD and PD onset, development and progression (Landrigan et al. 2005).

## Conclusion

Although a growing body of evidence suggests that the prevailing sporadic AD and idiopathic PD are fundamentally metabolic diseases with characteristic neurodegenerative processes possibly being caused by IRBS and metabolic dysfunction (Hoyer 2004; Defelice and Ferreira 2002; de la Monte et al. 2014; Chen et al. 2017; Bloom et al. 2018; Athauda et al. 2016; Schelp et al. 2017; Yang et al. 2018); the cause(s) and characterisation of these metabolic alterations are still unknown. One way to understand the metabolic etiopathogenesis of sporadic/idiopathic AD and PD is to explore the onset, development and time-course of insulin signalling dysfunction and glucose metabolism in the brain of animal models that mimic these human diseases. These models are generated by administration of particular neurotoxins, some of which could be considered as environmental toxins (e.g. MPTP as a recreational drug (Lau and Meredith 2003), and STZ as chemically related to nitrosamine-based food preservative (Tong et al. 2009)), and thus, more likely to mimic real-life exposure which might be associated with the increased risk for developing sporadic/idiopathic forms of AD and PD. In line with that, it is of utmost importance to detect toxicity targets of these neurotoxins at the molecular, cellular and tissue/regional level and assess where and to which extent do they overlap.

Sharing of the molecular mechanisms (mitochondrial damage) and co-expression of the molecular targets (IR, GLUT2, DAT) on membranes of particular neurons as well as astrocytes might be the underlying mechanism for insulin-related metabolic alterations present in both AD and PD non-transgenic models. Dysfunction in IR and its downstream PI3K/AKT/GSK3 signalling pathway seems to be a key pathology shared by the models (Table 1), occurring either as a primary toxic event, and, thus, expressed to a larger extent with consequently severe cognitive deficits in non-transgenic AD models, or as a collateral damage, expressed to a lesser extent and leading to less dominant cognitive impairment in non-transgenic PD models (Fig. 1). Based on that, IRBS could be considered a common metabolic pathology in the representative non-transgenic animal models of both AD and PD which, instead of being a cause, seems to be a common contributor to neurodegenerative processes. Further research is needed to provide in depth characterization of the brain insulin and dopamine signalling interaction in particular models at the molecular, cellular and regional level, for more successful animal-to-human data translation.
